# Successful Repair of Cor Triatriatum Sinistrum in Childhood: A Single-Institution Experience of Two Cases

**DOI:** 10.7759/cureus.24579

**Published:** 2022-04-29

**Authors:** Vishal V Bhende, Hardil P Majmudar, Tanishq S Sharma, Deepakkumar V Mehta, Amit Kumar, Jigar P Thacker, Gurpreet Panesar, Kunal Soni, Kartik B Dhami, Nirja Patel, Sohilkhan R Pathan

**Affiliations:** 1 Pediatric Cardiac Surgery, Bhanubhai and Madhuben Patel Cardiac Center, Bhaikaka University, Karamsad, IND; 2 Radiodiagnosis and Imaging, Pramukhswami Medical College and Shree Krishna Hospital, Bhaikaka University, Karamsad, IND; 3 Pediatric Cardiac Intensive Care, Bhanubhai and Madhuben Patel Cardiac Center, Bhaikaka University, Karamsad, IND; 4 Pediatrics, Pramukhswami Medical College and Shree Krishna Hospital, Bhaikaka University, Karamsad, IND; 5 Cardiac Anesthesiology, Bhanubhai and Madhuben Patel Cardiac Center, Bhaikaka University, Karamsad, IND; 6 Central Research Services, Bhanubhai and Madhuben Patel Cardiac Center, Bhaikaka University, Karamsad, IND

**Keywords:** congestive cardiac failure, left atrial membrane, pulmonary vascular disease, congenital heart disease, cor triatriatum

## Abstract

Cor triatriatum is a rare structural congenital cardiac anomaly in which one of the atria chambers is anatomically divided. If left untreated, cor triatriatum can eventually lead to heart failure. This case report describes our experience with two pediatric patients (a three-year-old girl and an 11-month-old male infant) who underwent surgical correction of cor triatriatum. Both patients underwent excision of the cor triatriatum membrane via cardiopulmonary bypass and had an uneventful postoperative recovery with good outcomes. Surgical repair of cor triatriatum sinister provides satisfactory short-term and long-term outcomes with a low risk of requiring additional intervention.

## Introduction

Cor triatriatum is a rare anatomic, congenital cardiac anomaly. The atrium is divided into two chambers by a membrane obstructing the blood flow in the left (cor triatriatum sinister) or the right atrium (cor triatriatum dexter), eventually leading to cardiac failure [1]. Cor triatriatum sinister involves obstruction of pulmonary venous drainage, pulmonary arterial and venous hypertension, and congestive cardiac failure. Pulmonary hypertension and congestive heart failure depend on intraatrial obstruction levels and related congenital anomalies. In this condition, the pulmonary veins drain into the proximal chamber located superoposterior, and the mitral valve and left atrial appendage reside in the distal chamber in the anteroinferior position. The embryologic basis of this condition is a topic of debate with researchers suggesting mal septation, mal incorporation, or entrapment during cardiac development as likely causes [1-4]. Lewis et al. first reported operative treatment of cor triatriatum in 1956 [5].

The definitive treatment of cor triatriatum is resection of the membrane using cardiopulmonary bypass (CPB) [1]. Most patients have associated congenital cardiovascular anomalies [6-8], and surgical outcomes depend upon the complexity of those associated cardiac anomalies [9,10]. This case report describes our experience with the surgical repair of cor triatriatum in a three-year-old girl and an 11-month-old male infant.

## Case presentation

Case 1

**Figure 1:**
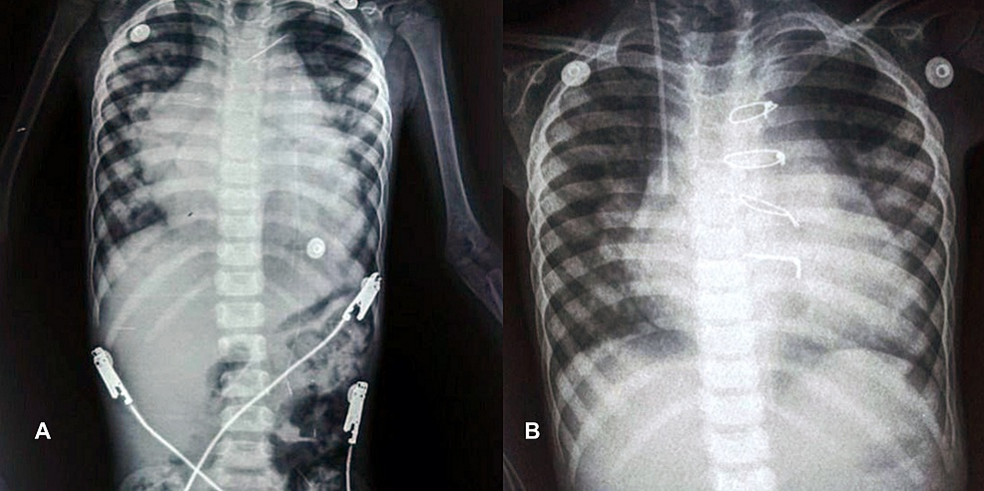
Preoperative (A) and postoperative (B) chest x-ray of the three-year-old girl

Our first case is a three-year-old girl weighing 9.5 kg who presented to our clinic. She had a history of multiple episodes of hospital admission for difficult breathing. On admission, she received intravenous milrinone for severe right ventricular dysfunction. She had intermittent fever managed with antipyretic medications. The patient was diagnosed with cor triatriatum, atrial septal defect (ASD), severe tricuspid regurgitation, moderate right ventricle dysfunction, and severe pulmonary arterial hypertension. As part of her cardiac workup, a chest x-ray was done showing cardiomegaly (Figures 1A, 1B).

Incidentally, she was diagnosed with sickle cell anemia via hemoglobin electrophoresis, and we planned for an intraoperative transfusion accordingly. We noted she had high pulmonary vascular resistance during her preoperative diagnostic cardiac catheterization with oximetry. We reduced her pulmonary vascular resistance by administering supplemental oxygen and sildenafil in increasing doses until the desired effect was achieved. She had febrile episodes with lymphocytosis and elevated c-reactive protein levels following her catheterization, so we deferred surgical correction.

Twelve days following admission, her laboratory values and clinical parameters were stable, and therefore she underwent surgical correction of her cor triatriatum. We excised the membrane in the left atrium and closed the ASD using an autologous pericardial patch.

To address her anemia, we conducted a controlled exchange transfusion of 300 mL of blood using a separate roller pump (60 mL/min for 10 min) with simultaneous infusion of prime volume at the same rate to avoid hypotension after cannulation. We performed a normothermic CPB with warm blood cardioplegia (maintained at >34°C). Her pH was maintained at 7.4, and her partial pressure of oxygen was >300 mmHg. We did not use a vasoconstrictor while on CPB.

The surgical procedure had no adverse events or hemolysis. She was weaned off the CPB with milrinone and adrenaline. We used modified ultra-filtration [11]. She was transferred to the surgical cardiac intensive care unit with inotropic support. On discharge, her hemoglobin level was 13.4 g/dL, and she was monitored via follow-up in our outpatient cardiac department.

Case 2

**Figure 2:**
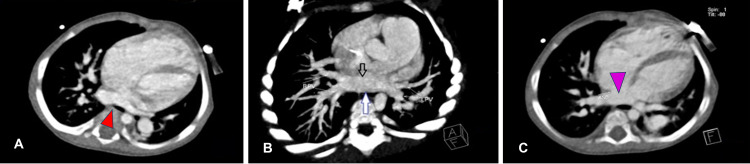
Contrast cardiac CT scan (axial view) of the 11-month-old boy (A) Axial image showing membrane of cor triatriatum (red arrowhead), left atrium, and ASD. (B) Axial maximum intensity projection showing membrane of cor triatriatum (black arrow) and posterior wall of cor triatriatum (blue arrow). (C) Large ASD between right and left atria (purple arrowhead). Abbreviations: CT, computed tomography; ASD, atrial septal defect.

**Figure 3:**
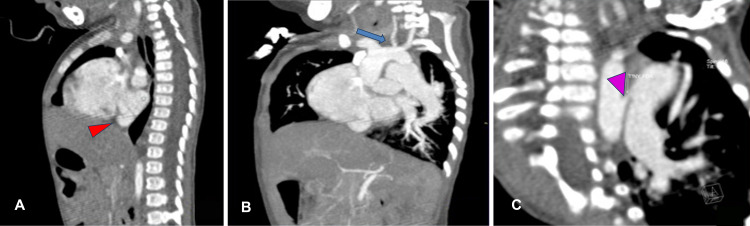
Contrast cardiac CT scan (sagittal and coronal view) of the 11-month-old boy (A) Sagittal reconstruction showing membrane of cor triatriatum (red arrowhead). (B) Oblique coronal reconstruction showing hypoplastic left common carotid artery (blue arrow). (C) Oblique coronal reconstruction showing tiny PDA. PDA: patent ductus arteriosus

An 11-month-old male infant weighing 4.88 kg presented to the pediatric cardiac outpatient department with a history of not gaining adequate weight and not establishing proper complementary feeding. Obstetric examination revealed a full-term male baby with spontaneous vaginal delivery and birth weight of 2.65 kg, and the baby had a good cry. The infant had no significant perinatal history or prior admission, and we noted uncorrected ankyloglossia on examination. The infant was admitted for a left complete irreducible inguinal hernia, and we incidentally diagnosed him with congenital heart disease. Two-dimensional echocardiography revealed partially absorbed pulmonary venous confluence or cor triatriatum with two atrial septal defects, shunting left to right with moderate patent ductus arteriosus. He also had moderate to severe pulmonary arterial hypertension with good biventricular function. Because the diagnosis of cor triatriatum was not confirmed, we obtained a dynamic cardiac computed tomography study (Figures 2A-2C, 3A-3C).

**Figure 4:**
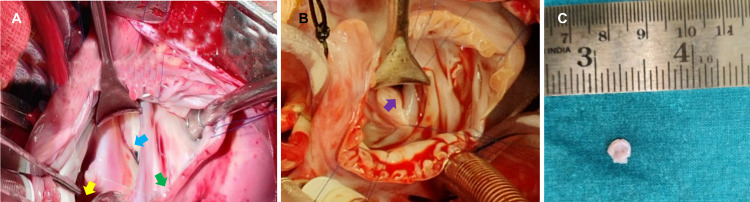
Intraoperative images of the three-year-old girl and 11-month-old boy (A) Case 1 - cor triatriatum is denoted by the green arrow, the mitral valve is denoted by the blue arrow, and the pulmonary veins are denoted by the yellow arrow. (B) Case 2 - purple arrow denotes the cor triatriatum sinister membrane in the left atrium. (C) Excised specimen of cor triatriatum sinistrum membrane from case 2 sent for histopathological examination.

Following his left inguinal herniotomy with uneventful recovery, we surgically corrected the cor triatriatum sinister by excising the membrane in the left atrium and closed the ASD using an autologous pericardial patch (Figures 4A, 4B). We also ligated the patent ductus arteriosus. The histopathology report from the excised specimen stated the tissue had two surfaces without any discernible lining and predominantly consisted of myocardial fibers and fibrous connective tissue (Figure 4C). The connective tissue was dense just below the surface with edema.

The patient also underwent correction of his ankyloglossia. His postoperative recovery was uneventful. He successfully fed for longer intervals than he could preoperatively due to the tongue-tie release. He was monitored via follow-up in our outpatient cardiac department. The intraoperative and postoperative parameters for case 1 and case 2 are represented below (Table 1).

**Table 1:** Intraoperative and postoperative parameters for case 1 and case 2 ACC: aortic cross-clamp; CPB: cardiopulmonary bypass

Parameters	Case 1	Case 2
ACC time (minutes)	47	76
CPB time (minutes)	72	117
Ventilator time (hours)	27	49
Hospital stay (in days)	21	14

## Discussion

**Figure 5:**
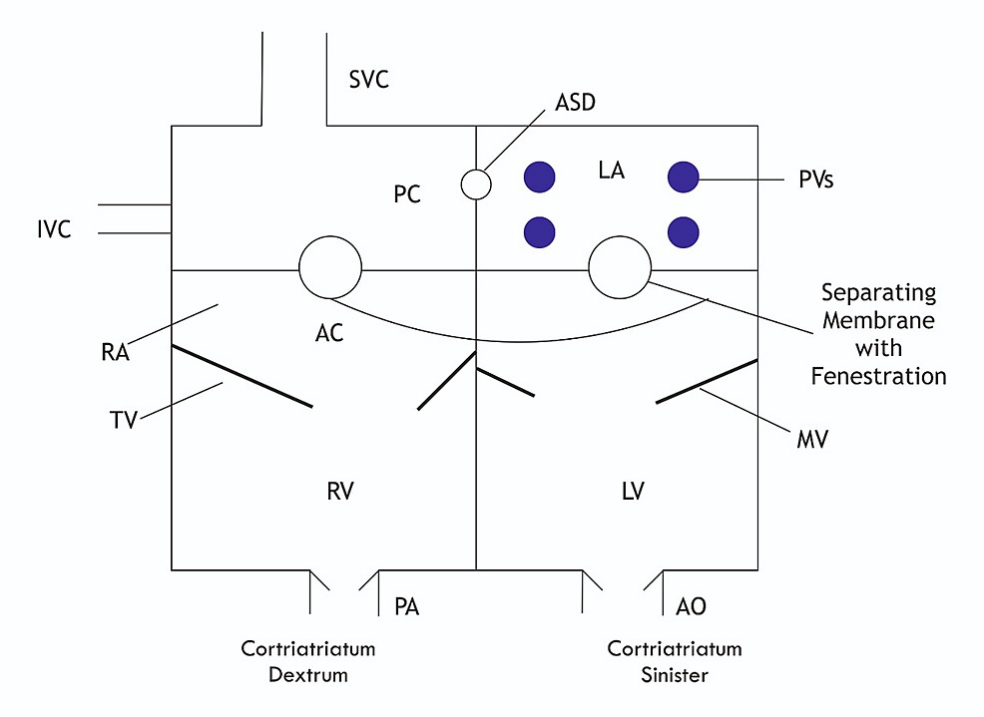
Diagram of cor triatriatum sinistrum and dextrum anatomy SVC: superior vena cava; IVC: inferior vena cava; RA: right atrium; LA: left atrium; PVs: pulmonary veins; PA: pulmonary artery; ASD: atrial septal defect; AO: aorta; AC: anterior chamber; PC: posterior chamber; RV: right ventricle; LV: left ventricle; TV: tricuspid valve; MV: mitral valve

Cor triatriatum, also called as triatrial heart, is one of the rarest congenital heart conditions as it accounts for only 0.1% of patients with congenital heart disease. This condition was first described by Church in 1868 in a first postmortem case in which a fibromuscular membrane divides the left atrium into two chambers [12]. Cor triatriatum sinister is a surgically correctable congenital cardiac anomaly as illustrated in Figure 5.

Echocardiography is critical for preoperative diagnosis, intraoperative evaluation, and postoperative follow-up after surgical repair [1,13]. As in our two cases, correction tends to occur while patients are very young, and the mean age of diagnosis will likely decrease as echocardiography becomes more widely available in clinical practices [7,9].

Cor triatriatum has a broad spectrum of presentations. Infants can present with shock, pulmonary edema, respiratory failure, and pulmonary hypertension in the most severe forms of this condition [14]. Older patients with cor triatriatum experience progressive mechanical obstruction, atrial fibrillation, or cardiovascular lesions [15]. The symptoms of this anomaly are usually deduced by the extent of hemodynamic obstruction and the probability of the presence or absence of interatrial communication. ASD and patent foramen ovale are typical concomitant defects in these patients. One series reported interatrial septal defects in up to 60% of patients [16]. As cor-triatriatum is a rare congenital cardiac anomaly and in our study both the patients had associated conditions that required to be tackled during the intraoperative period.

In case 1, since the HbS was > 30% before surgery, a preoperative exchange transfusion was planned as it promotes perfusion as well as helps to reduce perioperative sickling crisis and multiple-organ failure incidence. Exchange transfusion is beneficial in decreasing HbS and increasing preoperative HbA, hematocrit, and cellular oxygen delivery. Exposure to cold, stress, dehydration, infections, inflammatory cascades, hypoxia, and acidosis are some predisposing factors for sickling. These conditions are pretty common in cardiac surgery [17].

In case 2, The patient who underwent cor triatriatum repair had an associated tongue-tie detected in the pre-operative evaluation which was released using bipolar diathermy in the same sitting. This child was also diagnosed to have a left complete irreducible inguinal hernia and underwent a left inguinal herniotomy before cardiac surgical correction.

## Conclusions

Cor triatriatum is associated with several congenital cardiac abnormalities in most patients. Surgical repair is the definitive modality of treatment for symptomatic patients, and, as highlighted by our two cases, surgical treatment leads to satisfactory short-term and long-term outcomes. In addition to cor triatriatum repair in both the cases, the additional presence of sickle cell syndrome and release of tongue-tie in cases 1 and 2, respectively, in the perioperative period make it a unique and interesting combination with good results. Clinical presentation and the extent of complexity of cardiac anomalies seem to have had an adverse effect on the survival after repair.
